# New Pieces for an Old Puzzle: Approaching Parkinson’s Disease from Translatable Animal Models, Gut Microbiota Modulation, and Lipidomics

**DOI:** 10.3390/nu15122775

**Published:** 2023-06-16

**Authors:** Lorena Ortega Moreno, Ana Bagues, Vicente Martínez, Raquel Abalo

**Affiliations:** 1 Department of Basic Health Sciences, Faculty of Health Sciences, University Rey Juan Carlos (URJC), 28922 Alcorcón, Spain; 2 High Performance Research Group in Physiopathology and Pharmacology of the Digestive System (NeuGut-URJC), University Rey Juan Carlos (URJC), 28922 Alcorcón, Spain; 3 Associated I+D+i Unit to the Institute of Medicinal Chemistry (IQM), Scientific Research Superior Council (CSIC), 28006 Madrid, Spain; 4 High Performance Research Group in Experimental Pharmacology (PHARMAKOM-URJC), University Rey Juan Carlos (URJC), 28922 Alcorcón, Spain; 5 Department of Cell Biology, Physiology and Immunology, Universitat Autònoma de Barcelona, 08193 Barcelona, Spain; 6 Neuroscience Institute, Universitat Autònoma de Barcelona, 08193 Barcelona, Spain; 7 Centro de Investigación Biomédica en Red de Enfermedades Hepáticas y Digestivas (CIBERehd), Instituto de Salud Carlos III, 28049 Madrid, Spain; 8 Working Group of Basic Sciences on Pain and Analgesia of the Spanish Pain Society, 28046 Madrid, Spain; 9 Working Group of Basic Sciences on Cannabinoids of the Spanish Pain Society, 28046 Madrid, Spain

**Keywords:** animal models, constipation, dopamine, gastrointestinal, gut-brain axis, lipidome, microbiota, non-motor symptoms, Parkinson’s disease, probiotics

## Abstract

Parkinson’s disease (PD) is a severe neurodegenerative disease characterized by disabling motor alterations that are diagnosed at a relatively late stage in its development, and non-motor symptoms, including those affecting the gastrointestinal tract (mainly constipation), which start much earlier than the motor symptoms. Remarkably, current treatments only reduce motor symptoms, not without important drawbacks (relatively low efficiency and impactful side effects). Thus, new approaches are needed to halt PD progression and, possibly, to prevent its development, including new therapeutic strategies that target PD etiopathogeny and new biomarkers. Our aim was to review some of these new approaches. Although PD is complex and heterogeneous, compelling evidence suggests it might have a gastrointestinal origin, at least in a significant number of patients, and findings in recently developed animal models strongly support this hypothesis. Furthermore, the modulation of the gut microbiome, mainly through probiotics, is being tested to improve motor and non-motor symptoms and even to prevent PD. Finally, lipidomics has emerged as a useful tool to identify lipid biomarkers that may help analyze PD progression and treatment efficacy in a personalized manner, although, as of today, it has only scarcely been applied to monitor gut motility, dysbiosis, and probiotic effects in PD. Altogether, these new pieces should be helpful in solving the old puzzle of PD.

## 1. Introduction

Parkinson’s disease (PD) is the most frequent movement disorder and the second most common neurodegenerative disease, after Alzheimer’s disease [1]. With an incidence of 10–19 per 100,000 person-years [2], PD affects approximately 1% of the population over 60 years old [3], although approximately 10% of PD cases, known as early-onset PD patients, are diagnosed in their 30s [4]. Importantly, PD is the fastest-growing neurological disorder, and it is expected that by 2040 it will affect more than 12 million people worldwide [5,6]. Although the “shaking palsy” was first described by Parkinson in 1817, much of its pathophysiology is still unknown. Furthermore, current management is still purely symptomatic, despite all the efforts devoted to investigating new, more effective therapies [1]. Sadly, death occurs after an average of 15 years after diagnosis [6]. Therefore, this disease has an enormous impact not only on the individuals who suffer from it and their families, but also on the health system and society [7]. Not surprisingly, the economic burden of the disease is very large, estimated in 2017 as USD 51.9 billion in the United States alone [8].

PD is well known to be characterized by the irreversible loss of dopaminergic neurons in the substantia nigra pars compacta (SNpc) and their corresponding axon terminals in the striatum [9]. This results in several motor symptoms that are characteristic of PD, such as bradykinesia, muscular stiffness, resting tremors, akinesia, hypokinesia, and postural imbalance [7]. Unfortunately, by the time PD is diagnosed, which is based on these motor symptoms, at least 60–80% of dopaminergic neurons have already been lost in the SNpc [10].

**Figure 1:**
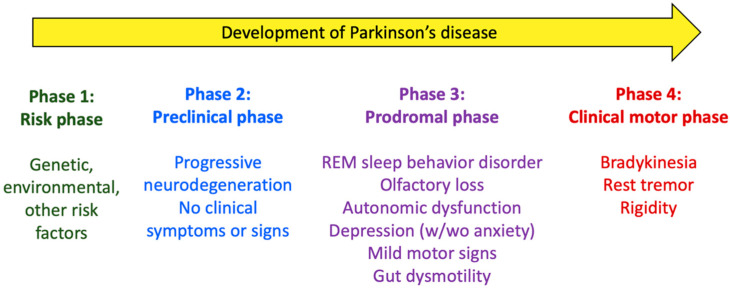
Phases of the development of Parkinson’s disease. Abbreviations: w, with; wo, without.

Therefore, it is believed that the disease starts well before the motor symptoms are clinically relevant. As shown in Figure 1, before diagnosis, PD develops slowly and continuously along four phases [1,11,12]: (1) the risk phase, in which different genetic, environmental, and other risk factors contribute to the development of the disease; (2) the preclinical phase, in which there are no clinical symptoms at all, but neurodegeneration occurs; (3) the prodromal phase, in which motor symptoms may occur but are only mild, whereas other non-motor symptoms are already obvious and impactful, including (not necessarily in this chronological order) altered REM (rapid eye movement) sleep cycle and circadian disruption, olfactory dysfunction (hyposmia), autonomic dysfunction affecting the cardiovascular and the gastrointestinal (GI) system, among others, and depression (with or without anxiety); and (4) the clinical motor phase, in which the typical PD motor symptoms are evident and lead to diagnosis.

Accordingly, the Movement Disorders Society (MDS) Research Criteria for Prodromal PD define the non-motor symptoms of PD as REM sleep behavior disorder (RBD), abnormal results of dopaminergic positron emission tomography (PET), abnormal quantitative motor testing, olfactory loss, constipation, excessive daytime somnolence, symptomatic hypotension, erectile dysfunction, urinary dysfunction, and depression with or without anxiety [13]. Clearly, PD can be considered a multiorgan disorder.

Current PD treatments try to compensate for the loss of dopamine (DA), mainly through supplementation of the DA precursor, L-DOPA, although other adjuvants may be used. However, these treatments are (moderately) successful only in reducing motor symptoms and cause significant side effects that reduce the quality of life of PD patients [14]. Nonpharmacologic therapies with some clinical benefit include surgery and deep brain electrical stimulation [15]. In addition, environmental enrichment, comprising physical exercise, cognitive stimulus, and social interactions, is being assessed as a possible way to achieve neuroprotection in PD [16].

However, thus far, no drug or technique has been approved as a neuroprotective or disease-modifying therapy for PD. Several reasons can explain the difficulties to find effective strategies to halt and, ideally, reverse PD. First, the animal models classically used in PD research are not optimal, and only recently some new models have been shown to be able to mimic, more properly, the progressive development of the disease and its impact on the body systems apart from the brain, as well as the etiopathogenic mechanisms involved [17]. Second, the pathogenic mechanisms themselves are not completely understood; therefore, it is difficult to establish which is the initiating causative mechanism of the disease, if there is only one. Indeed, PD patients are a greatly heterogeneous population, with differences in the progression of the disease (fast vs. slow), in the prevalence of motor and non-motor symptoms, and in the influence of genetic variants. This heterogeneity is difficult to mimic experimentally and may require the stratification of patients, according to their specific phenotype (deep phenotyping), to design personalized treatments [18]. Finally, reliable biomarkers of the disease, both for diagnosis and to determine the efficacy of the treatment in patients and animal models, are lacking.

In this review, we will provide first a brief overview of PD’s pathogenic features and the factors that are known to influence its development and severity, with special attention to the role of the GI tract and GI dysmotility. Then, the main animal models available to mimic PD will be described, highlighting the advantages and disadvantages of each one that may impact the translatability of the findings, particularly in reference to the GI system, and how GI motility has been evaluated in them. Thereafter, we will analyze the characteristics of gut microbiota in PD and the potential role of microbial dysbiosis on PD pathogenesis and PD-associated constipation. Finally, we will explore the potential diagnostic and prognostic usefulness of lipidomics as a new tool in preclinical and clinical research, including its possible use to evaluate the efficacy of gut microbiota modulating PD and PD-associated GI dysfunction.

## 2. Parkinson’s Disease: Pathogeny, Risk/Protection Factors, and Gut Involvement

Lewy bodies (LB), which consist of aggregated α-synuclein, and other aggregation-prone proteins are the pathophysiological hallmark of PD [19]. In addition to α-synuclein misfolding and aggregation, other cell and tissue alterations underlie the initiation and progression of PD, including mitochondrial dysfunction [20], altered protein clearance (involving ubiquitin-proteasome) [21] and autophagy-lysosomal systems [22], neuroinflammation (to which autoimmunity may contribute [23]), and oxidative stress [24,25]. Although the dopaminergic system is key to the pathogenesis of PD, non-dopaminergic, non-catecholaminergic, and non-motor systems are also involved [26]. Furthermore, it has been demonstrated that neurodegeneration affects neurons from several nuclei, such as the locus coeruleus, raphe nuclei, and the dorsal motor nucleus of the vagus (DMX), long before the SNpc [27,28], which partly explains the early occurrence of non-motor symptoms. Curiously enough, central protein aggregation seems to display an ascending gradient, originating at the bottom of the brain stem, expanding to the basal ganglia, and ending in the cerebral cortex, in a prion-like manner [29].

Aging (or “inflammaging,” the sustained systemic inflammatory state that develops with advanced age, as a result of the exposure to chronic stressors or the imbalance between proinflammatory and anti-inflammatory pathways, together with the immunosenescence phenomena [30]) is still the single most important risk factor for the development of PD, including its motor and non-motor symptoms [31].

However, PD is considered multifactorial, and other risk factors seem to contribute to its initiation and progression. Although heritable factors (mutations in around 10 identified genes) account for some PD cases, most of them (>90%) are sporadic, namely related to exposure to different environmental factors [32,33]. These include, for example, environmental toxicants, such as pesticides, herbicides, fungicides, polymers, and solvents [34,35,36], as well as heavy metals (e.g., iron, manganese, mercury, lead, copper), due to occupational exposure, contaminated food, medical products (including implants or dental restorations [37]), and environmental pollution. All these factors display synergistic effects when co-exposure occurs [38]. Intriguingly, despite the suggested triggering role of heavy metals in the development of PD [33], cisplatin was shown to inhibit filamentous aggregation of α-synuclein in solution [39], but the effects of this and other platinum-based antitumoral drugs in cancer survivors related to the appearance of PD have not been specifically determined.

Other environmental factors that may contribute to the pathogenesis of PD are those related to lifestyle [40], somewhat more disputed but probably also relevant to the current increasing rate of appearance of the disease. For example, chronic stress and vitamin D deficiency, associated with increased levels of cortisol and decreased levels of klotho (findings also encountered in PD), are thought to play a role in the pathogenesis of PD, favoring mitochondrial dysfunction, oxidative stress, insulin resistance, and neuroinflammation [41,42]. In contrast, physical exercise, which reduces psychological stress and helps to achieve healthy aging, might exert neuroprotective actions, through improvements of cortisol and klotho levels, as well as promoting metabolic and mitochondrial remodeling, improving the endocrine profile derived from the muscle and adipose tissue, with potential beneficial effects in PD [42]. Sleep disturbance and circadian disruption might be not only a chronic symptom of PD, but an additional risk factor for its development [43]. Thus, melatonin has been shown to inhibit α-synuclein assembly and to destabilize fibrils, which suggests a preventive role of this hormone against α-synuclein cytotoxicity and neurodegeneration [44]. In addition, the glymphatic system, the system that removes extracellular brain solutes, does so mainly during sleep (optimally, during REM sleep); therefore, sleep impairment may weaken the activity of this system, which may contribute to α-synuclein accumulation and, consequently, dopaminergic neuron loss [43]. Interestingly, together with physical exercise, smoking and caffeine/coffee intake (despite the general idea that acute and chronic intake of caffeine is associated with less sleep or more disturbed sleep [45,46]) are considered neuroprotectant factors against the development of PD [47].

Lastly, the fact that early pathological changes are seen in the gut and the olfactory system has recently led to the hypothesis that the triggering event of PD could be a toxic or infectious agent, “transmitted” to the central nervous system (CNS) after coming in contact with the olfactory nerve terminals in the nasal mucosa or with the vagus nerve terminals in the upper GI tract [47].

Indeed, among the non-motor symptoms that affect PD patients during the prodromal phase, at least 10–20 years before diagnosis, GI dysmotility, particularly constipation [48], is one of the most common and bothersome symptoms [49], although it is also relatively unspecific and its usability as a single biomarker of the disease is low. Importantly, even if GI dysmotility, particularly constipation, cannot be used as a biomarker for diagnosis, it is a symptom encountered in most PD patients, which may become even more severe due to antiparkinsonian and antidepressant treatments [50].

The presence of GI manifestations (constipation, dysphagia, drooling, gastric dysmotility) much earlier than the onset of PD motor symptoms suggests that α-synuclein deposition may start in the enteric nervous system (ENS) and only later propagate to the CNS [51,52]. Indeed, in accordance with the so-called Braak’s staging hypothesis [53], LB can be found in the enteric neurons many years before the onset of motor symptoms in PD patients [54]. In general, it is believed that constipation and gut mucosa inflammation are associated with the loss of neurons and glial cells in the ENS, although more robust evidence of this has recently been claimed [55]. Indeed, the role of the ENS in PD development (whether it is a gateway from which the disease is triggered or whether it might be a source of solutions for it) is not clear [56].

Furthermore, it has been suggested that an altered gut microenvironment (gut dysbiosis) is a potential trigger of PD in a genetically susceptible host [57]. This may increase intestinal permeability, aggravate neuroinflammation, and speed up the neurodegeneration of enteric neurons. Therefore, the restoration of the gut microbiome in PD patients has been proposed as a strategy not only to improve the GI symptoms but to delay the clinical progression of the disease [58,59,60] (see below).

Nevertheless, it should be considered that GI motility disturbances themselves alter microbial composition, making it difficult (if not impossible) to ascertain which one of these factors precipitates PD in patients. Preclinical research might be particularly important in giving light to this issue.

## 3. Gastrointestinal Motor Function in Animal Models of Parkinson’s Disease

Animal models of PD permit an approach to study this disease, providing a further understanding of the pathological mechanisms and, ultimately, the development of drugs for the prevention and treatment of its symptoms. Animal models can be performed in nonvertebrate (such as the fruit fly) or vertebrate animals. The latter are necessary for functional and behavioral analysis, thus increasing translatability to humans [61].

Of all vertebrates, rodents are preferred because they have a significant similarity in the cortico-basal ganglia-thalamocortical loops and their behavioral corresponding functions to humans. Additionally, rodents present similar motor function deficits after lesions to the nigrostriatal dopaminergic neurons and analogous motor responses to DA replacement therapy [61]. These facts, added to the lower costs and fewer ethical issues that accompany the use of rodents compared with other vertebrates (cats, dogs, or primates), make them the most widely used animal model to mimic PD, and they will be succinctly reviewed in this section. If available, the main functional and structural alterations of the GI tract (including gut microbiota) described in these PD models will be highlighted.

### 3.1. Rodent-Based Animal Models of PD

There are many different types of in vivo PD models, which fall mainly into two main categories: the toxin-induced models and the genetic ones. However, none is capable of reproducing the entire phenotype of PD, and they lack some neuropathological or behavioral features [62].

In the following section, a brief description of the genetic and neurotoxin models is given. An extensive review is beyond the scope of this paper and readers are referred to more extensive reviews in each section.

#### 3.1.1. Genetic Models

Transgenic animal models have been developed after the finding of different human mutations implicated in familial cases of PD. Indeed, mutations in specific genes that cause mitochondrial dysregulation and energy disturbances have been shown to cause PD [63].

The first gene to be unequivocally related to PD was the α-synuclein gene (SNCA) [64]. Since then, up to 15 causal genes and more than 25 genetic risk factors have been identified. The most common ones, which have been modeled in rats and mice, are SNCA (α-synuclein, PARK1 and 4), PRKN (parkin RBR E3 ubiquitin protein ligase, PARK2), PINK1 (PTEN-induced putative kinase 1, PARK6), DJ-1 (PARK7), and LRRK2 (leucine-rich repeat kinase 2, PARK8) [65].

These models, although useful in the understanding of the molecular and biochemical pathways implicated in PD, do not completely reproduce the pathological and behavioral phenotype of PD in humans, and some do not display nigrostriatal degeneration, striatal dopaminergic depletion, nor motor symptoms (for further review, see [65,66]). However, most interestingly, in a recent systematic review, GI dysfunction was shown to be the most reproducible non-motor phenotype found across all genetic rodent models evaluated [17].

In this regard, the substitution of alanine to threonine at position 53 of the α-synuclein protein (A53T) was identified as the cause of a severe autosomal dominant trait of Parkinsonism. The A53T transgenic mice model, possibly the most frequently used genetic model, has been shown to have constantly delayed whole GI transit and colonic propulsion throughout different studies [67,68,69,70]. Other genetic models, such as the A30P, another mutant form of α-synuclein, or Mitopark model, which consists on the disruption of mitochondrial transcription factor TFAM, have also shown important GI transit alterations through different tests [71,72]. For a more extensive review, see [17].

Additionally, how genetic alterations modify the microbiome and, in turn, can increase the risk of PD has also been investigated. For example, Singh et al. (2020) demonstrated that DJ-1 knockout mice showed alterations in the microbiome, with alterations in the innate immune system, an increase in inflammatory proteins, and increased inflammatory genes in the midbrain of these mice [73]. Similarly, in the A53T mouse model, alterations in the microbiota when compared to wild-type littermates were observed much earlier than motor symptoms [74].

#### 3.1.2. Neurotoxin-Based Models

Models generated by the administration of neurotoxins are the most widely used to study PD, because they can be easily developed, and different treatments can be compared. Table 1 summarizes the main characteristics of the most widely used neurotoxin-based models.

##### 6-Hydroxydopamine

6-hydroxydopamine (6-OHDA) was the first toxin to be administered to generate an animal model of PD [75]. 6-OHDA is a structural analogue of catecholamines. Since it is a hydrophilic molecule, it does not cross the blood–brain barrier (BBB); therefore, it must be directly administered in the substantia nigra (SN), the medial forebrain bundle (MFB), or the striatum, where it enters the neurons through the DA and noradrenaline (NA) transporters [61]. The site of administration depends on the magnitude and characteristics of neurodegeneration that are being sought. Commonly, unilateral administration is applied, because bilateral administration can cause aphagia, adipsia, and bilateral motor deficits; nevertheless, some studies have performed a bilateral injection to study changes in spatial memory and recognition [76].

This model has been a valuable tool to understand the pathophysiology of PD. It provides an economical and easy method to study the disease in rodents, where it targets specific catecholaminergic pathways. The death of the dopaminergic bodies of the neurons activates the glia, which is also implicated in the pathogenesis of the human disease, contributing to the proinflammatory state and, in turn, increasing neuron death [65].

When injecting 6-OHDA, motor dysfunctions are observed that can be evaluated using different tests. The classical test used in this model has been the Ungerstedt motor or rotational test [75], although alterations in limb asymmetry have also been associated with unilateral dopaminergic deficits, and locomotor or sensorimotor deficits have also been observed [61].

As drawbacks, this model does not mimic the multisystem pathology of PD, the progressive nigrostriatal degeneration, or the formation of characteristic LB-related pathology [76]. However, GI dysmotility has been successfully replicated, as shown, among others, with radiographic methods [77].

##### MPTP

MPTP (1-methyl-4-phenyl-1,2,3,6-tetrahydropyridine) was discovered in the 1980s as a contaminant in “synthetic heroin”. Drug addicts who consumed drugs with this toxin developed a severe parkinsonian syndrome [78].

MPTP is a lipophilic compound that can cross the BBB. In the brain, it is metabolized to an analogue of DA (1-methyl-4-phenylpyridinium, MPP+) that is taken up into the dopaminergic neurons by the DA transporter. This toxin was initially used in primates, which developed all the PD motor symptoms seen in humans. Thereafter, it has also been administered to minipigs and mice [79].

The effect of MPTP can vary considerably depending on the route of administration, dose, and strain of mouse, which could explain, for instance, why the finding of LB is not consistent across studies. High doses of MPTP have been shown to induce locomotor disturbances; although possibly due to peripheral neurointoxication, they are seen acutely only after MPTP administration. Sensorimotor deficits have been observed in MPTP animals and are reproduced weeks after treatment; for review, see Meredith et al. (2006) [80]. Interestingly, MPTP may cause GI dysmotility, as shown both in mice [81] and in macaques [82].

##### Pesticides

Several epidemiological studies have demonstrated the association between pesticide exposure, rural residence, and an increased risk of PD [83]. Of these pesticides, rotenone and paraquat have been used to develop PD animal models.

(a) Rotenone

Rotenone is a naturally occurring isoflavone widely used as an herbicide and pesticide. The rotenone PD model was first introduced by Ferrante et al. (1997) [84]. Although different administration routes have been used, rotenone can be systemically administered because it easily crosses biological membranes, due to its lipophilic nature, and it does not depend on DA transporters to enter the cytoplasm of the cell. Rotenone-associated side effects include cardiovascular toxicity and nonspecific cerebral damage [84,85].

The administration of rotenone induces most of the motor symptoms seen in PD: catalepsy, akinesia, reduced locomotion, postural instability, and shaking of the paws. Additionally, it also causes SNpc dopaminergic neuron loss, nigrostriatal dopaminergic denervation, LB-like inclusions containing α-synuclein and ubiquitin, and GI motor alterations [86,87]. Another advantage of this model is the slow neuronal degeneration it induces, mimicking more precisely the progression of the disease in humans. Therefore, it is useful for the study of neuroprotective agents [65,85].

As cons, this model induces high mortality in rodents and the nigrostriatal dopaminergic neuron damage seems to be very variable in rats, possibly dependent on the strain used. In this regard, Sprague Dawley rats seem to be particularly resistant, whereas Lewis rats are more susceptible [85].

(b) Paraquat

Paraquat is one of the most frequently used pesticides worldwide. It is an MPP+ structural analogue, which crosses the BBB and accumulates in dopaminergic neurons using the DA transporter to enter the neuron.

The methodologies used in paraquat-based models are diverse. In rodents, paraquat is administered systemically (usually, subcutaneously, intraperitoneally, or orally), and the dose and duration of administration are also extremely heterogeneous; therefore, the behavioral and phenotype results differ across studies with regard to striatal dopaminergic depletion and motor deficiencies [88,89]. To induce more robust nigrostriatal dopaminergic degeneration, paraquat has been co-administered with maneb, a manganese polymer used as a fungicide in agriculture [90,91].

When administering paraquat at low doses, it has been shown to cause an upregulation of α-synuclein in wild-type mice [92] and to increase markers of α-synuclein aggregation in the ENS in a transgenic model of synucleinopathy [93].

Recently, a new exposure paradigm was developed in which paraquat was administered at low doses through mini osmotic pumps. This administration pattern led to nigrostriatal dopaminergic neurodegeneration, characterized by a 41% significant loss of dopaminergic neurons in the SNpc, a significant decrease of 18% and 40% of DA levels in the striatum at weeks 5 and 8, respectively, and a significant decrease in motor performance in the rotarod and open field tests [94].

Interestingly, the joint intragastric administration of low doses of paraquat and lectin in Sprague Dawley rats induced a reduction in dopaminergic neurons in the SNpc with sensorimotor dysfunction, without any rotational behavior [95]. Importantly, synucleinopathy started in the myenteric neurons and was transmitted via the vagal nerve to the DMX before it reached the SNpc. Furthermore, this temporal pattern of events supports Braak’s staging hypothesis at the functional level, with gastric dysmotility developing first and impairment of motor functions afterwards. Importantly, vagotomy prevented the development of central synucleinopathy and motor symptoms. In addition, the model responded positively to treatment with L-DOPA and was developed in a relatively short period of time (2 weeks) [95]. Despite its noticeable translatability, to our knowledge, alterations of intestinal motility and gut microbiota have not yet been studied in this model.

##### Other Neurotoxins

Other substances have been used to induce damage to the dopaminergic neurons in the substantia nigra, such as lipopolysaccharide (LPS), thrombin, or α-synuclein protein injection (for review, see [96]).

LPS is the main component of the membrane of the Gram-negative bacteria. It activates the toll-like receptor (TLR)-4 and causes an important inflammatory response. The first published article that injected LPS in the nigrostriatal pathway of rats observed an important and long-term degeneration of the dopaminergic neurons of the substantia nigra, without affecting the serotoninergic or GABAergic neurons, in addition to an important macrophage/microglial activation [97].

Other studies have injected different concentrations of LPS directly in the striatum, causing neurodegeneration in the substantia nigra of rats [98] and mice [99], inflammatory response, oxidative stress, and activation of the TLR/NF-KB (nuclear factor-kappa B) pathway, with motor alterations [100].

The importance of the compound to this review is that it can also induce inflammation within the olfactory bulb and alterations in the colonic epithelial barrier function with increased mucosal vasculature, which might facilitate immune activation, the development of local inflammation, and even the development of dysbiotic states [101]. Furthermore, systemic LPS induces GI dysmotility [102,103], suggesting a possible connection between a prolonged exposure to this toxin (i.e., due to dysbiosis) and PD prodromal GI symptoms.

### 3.2. Evaluation of GI Motor Function in PD Models

Relatively few studies have analyzed the alterations of GI motor function in PD rodent models, and they have used different procedures, as briefly described here.

Very simple analyses, such as the study of changes in fecal pellet output (number per time unit, wet and dry weight, % water content), have been performed [104], but also specific in vivo and in vitro analyses of GI motor function have been used, such as whole gut transit, evaluated as the time it takes for a marker (e.g., carmine red or methylene blue) to appear in the feces after it has been intragastrically administered [67].

Gastric emptying rate and upper GI transit generally has been evaluated using invasive methods that have also been applied in PD models: the animal is gavaged a marker, which is allowed to travel distally for 20–30 min, and then the animal is euthanized and the remaining contents in the stomach and the % length of the intestine traveled by the front of the marker are measured [81,104].

In addition, noninvasive, radiographic techniques have been used, allowing for several measures to be employed along PD progression [77]. In this case, barium is gavaged, and serial X-rays are taken and semiquantitatively analyzed [105].

Another noninvasive method to measure GI motor function is the breath test. In this case, a substance labeled with ^13^C, such as octanoic acid, is orally administered to the animal, and the concentration of ^13^CO_2_ in exhaled air is measured. This test has been used to analyze gastric emptying in PD models [106]. Since this test is also used in PD patients [107], it is of obvious translatability.

To accurately evaluate changes in gastric motor function, strain gauges sutured to the gastric corpus and antrum have been used to record tone and contractile responses to drugs in vivo [95,108].

To evaluate specific colonic propulsion in vivo, the bead expulsion test has been used, in which a bead is inserted in the colon under light anesthesia, and the time taken from bead insertion to expulsion is measured [67]. Additionally, the colon may be cannulated in vivo after laparoscopy and its peristaltic activity in response to intraluminal fluid infusion recorded [109].

Peristaltic activity of colonic segments from control and PD animals has also been evaluated in organ bath experiments in which the frequency of peristaltic contractions as well as changes in longitudinal muscle shortening and intraluminal pressure were recorded [110]. To our knowledge, no study in PD models has used Dmaps to represent peristaltic activity or bead expulsion as the changes in diameter along the colonic segment throughout time, although some researchers have specifically evaluated the involvement of dopaminergic receptors in rat colonic motility using this method [111].

Finally, organ bath contractility studies of colonic strips have evaluated the responses to electrical field stimulation (which induces neurotransmitter release) in the absence or presence of different neural antagonists/blockers, allowing the identification of neurotransmission pathways specifically altered in the PD model [112].

Importantly, alterations of GI motor function in the PD models should be accompanied by pathological changes. In this regard, a recent study used immunohistochemical methods on whole-mount preparations to evaluate neuronal populations within the SNE in different environmental and genetic PD models, demonstrating the presence of a model-dependent enteric neuropathy [113].

## 4. Intestinal Microbiota and PD

The gut microbiota consists of a wide range of microorganisms (bacteria, archaea, fungi, and viruses), with an enormous and flexible gene pool, that actively interacts with the host and is key for GI homeostasis and numerous extra-digestive functions [114]. Gut dysbiosis is the alteration in structural and/or functional configuration of the gut microbiota causing, primarily, disruption of gut homeostasis, but having also extensive extra-intestinal effects [115,116]. Compelling evidence indicates that gut microbiota and microbial metabolites affect the ENS and the CNS via the so-called microbiota–gut–brain axis.

Evidence accumulated during recent years suggests that PD might be associated to a state of gut dysbiosis. Although divergent findings have hindered PD from being linked to the microbiome as a whole, most studies have reported relevant correlations between gut dysbiosis and PD clinical features, including disease severity, disease duration, motor symptoms, and non-motor symptoms as well as with medication effects (for review, see [117,118]). Nevertheless, the studies performed so far, taken together, demonstrate remarkable microbial discrepancies, particularly at the family and genus levels. This high variability has been associated to intra- and inter-subject factors such as microbial colonization at birth, gender, diet, age, and medications as well as to experimental design and methodological differences [117,118]. Therefore, the existing data, although indicating a potential association, preclude the confirmation of a causal relationship between dysbiosis and PD. Thus, it cannot be concluded if the observed gut microbial changes are a cause or an effect of PD. Importantly, dysbiosis has also been observed in different PD animal models, thus reinforcing the view that the coexistence of PD and dysbiosis is not a casual finding [119,120,121,122].

Early indication of an association between microbiota and PD comes from studies showing a strong association between PD and small intestinal bacterial overgrowth (SIBO), which affects approximately 50% of PD patients [123,124,125,126]. Confirming this association, the correction of SIBO with an antibiotic resulted in an improvement of GI symptoms along with an improvement in motor fluctuations [123,124]. Furthermore, it was observed that occurrence of SIBO was associated with more severe motor fluctuations (particularly “delayed on” and “no on”) of PD [124]. This is in line with epidemiological data showing that GI infections are associated with an elevated risk of PD [127,128]. Further evidence linking PD and intestinal microbiota was generated from animal models of the disease. First, in an α-synuclein transgenic PD mouse model, transplanted fecal microbiota from PD patients exacerbated motor dysfunction to a significantly greater degree than microbiota from healthy individuals [129]. Second, although loss-of-function mutations in PTEN-induce putative kinase 1 (PINK1) can cause PD in patients [130], PINK1-knockout mice do not develop PD-like symptoms and were reported to be generally healthy. This might be related to the fact that these mice were raised under germ-free conditions and thus lacked gut microbes. Indeed, infection of young PINK1-knockout mice with Gram-negative bacteria that cause mild intestinal symptoms was sufficient to trigger PD-like symptoms later in life [131].

From these observations, specific studies have addressed gut dysbiosis during PD aiming to characterize in detail the microbial changes present in PD patients. Overall, gut microbiota concentrations were reduced in PD patients when compared to those from age-matched healthy controls, affecting particularly the short chain fatty acid (SCFA)-producing bacterial groups [132,133]. This contrasts with the above-mentioned early hypothesis associating PD and SIBO and reinforces the idea that microbial changes during PD should be regarded as a phenomenon of dysbiosis, implying the proliferation and reduction of specific bacterial groups. Changes in biodiversity were also observed, particularly as it relates to β-diversity (similarity or dissimilarity between two or more sets of microbial communities) [118,133,134,135,136,137,138,139,140]. According to initial studies, α-diversity [the number (“richness”) and distribution (“evenness”) of bacteria within a single population or community] was not altered during PD, suggesting that abundance differences are more associated with PD than differences in microbiota diversity [133,135]. However, against this opinion, recent studies indicate a significant increase in α-diversity in PD patients vs. healthy controls [118,136,139].

Several metagenomic studies have helped to characterize the microbiota of PD patients. These studies revealed significant changes at different taxonomic levels, including both increases and decreases in relative abundance during PD vs. healthy controls (see Table 2 for a summary of changes at the phylum and family levels, and Table 3 for a summary of changes at the genus level). However, very often, contradictory results can be found [117,141,142]).

Despite the high heterogeneity reported, a recent study suggests that the main microbial changes in PD patients might be summarized in three clusters of co-occurring microorganisms [143]: a first cluster composed of opportunistic pathogens, phyla Bacteroidetes and Actinobacteria, which were overrepresented; a second cluster, composed of SCFA-producing bacteria, belonging to the phylum Firmicutes, all of which were reduced; and, finally, a third cluster composed of carbohydrate-metabolizing microorganisms, phyla Actinobacteria and Firmicutes, all of which were elevated.

Microbial changes have also been associated to the clinical phenotypes of PD, and some correlations with disease duration/stage, severity, motor symptoms, and non-motor symptoms have been established (for review, see [117]). It has been suggested that patients with marked dysbiosis may progress faster than those without it [159]. Overall, increased levels of *Clostridium coccoides* and *Lactobacillus gasseri* (currently, *Lacticaseibacillus gasseri*) were reported to be associated with the onset of PD and advanced-stage PD, respectively [157,160]. Moreover, low counts of *Bifidobacterium*, *Bacteroides fragilis*, and *Clostridium leptium* have been associated with the worsening of some PD symptoms [161]. Similarly, a direct connection was observed amid the amount of Enterobacteriaceae and the severity of gait difficulty, inflammation, and postural instability in PD [145,162]. A recent prediction model suggests that decreases in SCFA-producing genera (*Fusicatenibacter*, *Faecalibacterium*, and *Blautia*) as well as an increase in mucin-degrading genus (*Akkermansia*) predicted accelerated disease progression [159]. In any case, after onset of the disease, the gut microbiota seems to contribute significantly to PD progression and symptom severity. Therefore, the characteristics of the microbiota have been suggested as a potential biomarker to differentiate patients with rapidly and slowly progressing PD pathology [161].

Besides bacteria, other components of the microbiota might be relevant in PD. Bacteriophage components must be included in the concept of gut microbial dysbiosis. In this sense, it has been shown that phages interfere in the normal processes of DA production and intestinal permeability, two vital factors associated with early-stage symptoms of PD. Bacteriophages, which have previously been suggested to cause alterations in microbiota composition, lead to increased intestinal permeability and trigger persistent inflammation [163,164]. Alterations to certain bacteriophages in PD patients have also been reported, wherein a significant decrease of *Lactococcus* spp. is accompanied by over-representation of lytic *Lactococcus* phages [155]. The depletion of *Lactococcus* spp., as an important DA producer and regulator of intestinal permeability, may be related to PD progression [165]. No differences in fungal abundance have been observed in PD patients vs. healthy controls, but large-scale prospective studies are still needed. The potential role of other components of the microbiota, especially the virome, also deserves further studies [138,166].

### 4.1. Underlying Mechanisms Associating Intestinal Dysbiosis and PD

As mentioned above, evidence collected during the last decade supports the existence of a functional communication system between the gut, the gut microbiota, and the brain, the so-called microbiota–gut–brain axis [167]. Three major mechanisms underlie the complex regulation of the microbiota–gut–brain axis: inflammation and immune-related reactions, neuroendocrine mechanisms, and vagal reflexes [167]. These mechanisms involve numerous neuro-immuno-endocrine mediators of endogenous as well as microbial origin that are able to operate in a bidirectional manner, thus ensuring the maintenance of GI homeostasis. Accordingly, the gut microbiota can modulate brain function and activity, and, in turn, the CNS modulates intestinal functions as well as the gut microbiota.

**Figure 2:**
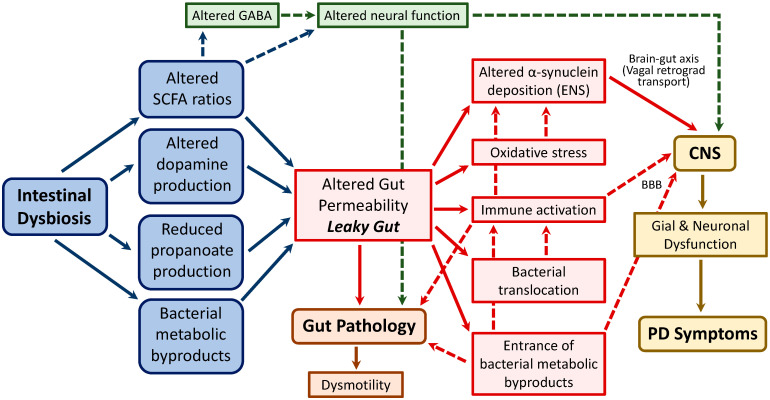
Schematic representation of the main pathophysiological mechanisms linking intestinal microbiota and PD. Dysbiosis is associated with altered patterns of microbial metabolism, including, as relevant for PD, altered ratios in the production of short-chain fatty acids (SCFA), changes in dopamine and propanoate production, and generation of different abnormal metabolic byproducts (for instance, increased levels of bacterial-derived lipopolysaccharides or toxins). All these factors lead to the alteration of gut permeability, generating a pathophysiological state known as “leaky gut.” The increase in permeability facilitates the entrance of immunogenic products from the intestinal lumen, including bacteria (bacterial translocation), bacterial components, and their metabolic byproducts. These factors interact among them in a positive manner, inducing metabolic changes within the intestinal cells (such as alterations in oxidative stress processes) and a state of immune activation. Altogether, these factors facilitate the altered deposition of α-synuclein in the enteric nervous system (ENS). Enteric α-synuclein can be retrogradely transported via the vagus nerve (brain–gut axis), in a prion-like manner, to the central nervous system (CNS). Within the CNS, α-synuclein will initiate the cascade of events that leads to glial and neuronal dysfunction, as a base for the neurological symptoms that characterize PD. Additionally, immune mediators and bacterial metabolic byproducts can also reach the CNS through alterations of blood–brain barrier (BBB) permeability, thus contributing to the glial and neuronal dysfunction. Moreover, the pathophysiological alterations present within the GI tract contribute to the development of gut pathology, which, in PD patients, is characterized by the presence of dysmotility resulting in constipation. Additionally, altered bacterial metabolism due to the dysbiotic state might produce alterations in the synthesis of neurotransmitters, such as GABA, contributing to the neural dysfunction.

The mechanisms by which intestinal dysbiosis may induce or promote PD pathology have not been completely elucidated. Overall, it is believed that dysbiosis can lead to increased gut mucosal permeability and inflammation, thus potentiating endotoxin exposure and the translocation of microbiota and microbial metabolites or byproducts. Moreover, all these factors interact in a self-maintenance manner, perpetuating and exacerbating the proinflammatory and dysbiotic state of the gut. As a consequence of these alterations, impaired SCFA balance and/or the induction of oxidative stress are considered key factors triggering α-synuclein aggregation within the ENS and its subsequent vagal translocation to the CNS, leading to the characteristic neurological alterations of PD [29,58,117,153,168,169,170,171,172] (Figure 2). Indeed, as mentioned above, intestinal inflammation, enhanced permeability, and dysmotility (including delayed gastric emptying and constipation) are common findings in PD patients [171,173,174,175], and α-synuclein aggregation has been observed in the GI tract long before the appearance of PD dysfunctions [176].

In this context, the reduction in *Prevotella*, consistently reported in PD patients (see Table 3) might be particularly important since it has been associated to a reduction in the levels of neuroactive SCFA, folate, and thiamine biosynthesis [177] as well as to a reduction in mucin biosynthesis, related to alterations in intestinal permeability (increased permeability leading to a state of “leaky gut”). In particular, SCFA are bacterial metabolites involved in the regulation of intestinal motor function, and a reduction in SCFA-producing bacteria may be key to the occurrence of constipation and other dysfunctions of the GI tract, including in the prodromal phase of PD [178]. The apparent importance of microbial SCFA metabolism in PD justifies the interest in lipidomics, as a new approach to the study of pathways and networks of cellular lipids, for the identification of microbial-related biomarkers of PD and its GI complications (see Section 5).

Recent data suggest also that microbial changes with an impact on the metabolism of histidine (enhanced degradation), proline (enhanced biosynthesis), isopropanol (enhanced biosynthesis), and pyruvate (reduced fermentation) might lead to a final state of reduced propanoate biosynthesis, enhancing intestinal permeability and thus facilitating the entrance of other bacterial metabolites, such as glutamate or isopropanol, which, upon arrival to the CNS, might exert detrimental effects on neuronal function [144].

### 4.2. Microbiota-Based Therapies for PD

Currently, there is no optimal treatment for PD, and available therapies are mainly symptom-based. According to the evidence implicating the gut microbiota in the pathogenesis and symptom progression of PD, microbiota-based therapies have emerged as an attractive approach for PD management. Following this, the use of probiotics and fecal transplantation have been particularly studied.

Studies in PD animal models suggest that probiotic treatment with several bacterial groups, including different mixtures of *Clostridium butiricum*, *Lactobacillus rhamnosus* (currently, *Lacticaseibacillus rhamnosus*), *Lactobacillus plantarum* LP28 (currently, *Lacticaseibacillus plantarum*), *Bifidobacterium lactis*, *Bifidobacterium longum*, *Lactococcus lactis,* and *Lactobacillus acidophilus* (currently, *Lacticaseibacillus acidophilus*), might exert neuroprotective effects with potential clinical relevance [179,180,181]. However, the translational value of these observations is still unclear. An early clinical study showed that a probiotic containing *Lactobacillus casei Shirota* (currently, *Lacticaseibacillus casei Shirota*) ameliorated constipation, bloating, abdominal pain, and the sensation of incomplete emptying in PD patients, thus suggesting an overall improvement in GI motility [182]. A recent clinical trial indicates that consumption of a probiotic, containing *Lactobacillus acidophilus* (currently, *Lacticaseibacillus acidophilus*), *Bifidobacterium bifidum*, *Lactobacillus reuteri* (currently, *Lacticaseibacillus reuteri*), and *Lactobacillus fermentum* (currently, *Lacticaseibacillus fermentum*), for a 12-week period had beneficial effects on PD patients, as evaluated by the Unified Parkinson’s Disease Rating Scale (UPDRS) [183]. Similarly, a recent pilot study assessing the efficacy of *Lactobacillus plantarum* (currently, *Lacticaseibacillus plantarum*) (PS128) also reported an improvement in UPDRS motor score and quality of life of PD patients [184]. Nevertheless, to date, the clinical data available are too limited, and additional, systematic studies are necessary. As of today, 12 clinical trials assessing the efficacy of different probiotic treatments on PD are registered, with different status of progress (ClinicalTrials.gov Identifiers: NCT04140760, NCT03377322, NCT04451096, NCT03968133, NCT04293159, NCT04389762, NCT02459717, NCT04722211, NCT04871464, NCT05173701, NCT05146921, and NCT01536769). Of these, only five trials have been completed; so far, only four of them (NCT04389762, NCT04451096, NCT03377322, and NCT02459717) have released the results obtained [184,185,186,187], whereas the fifth one (NCT01536769) has not. A recent meta-analysis analyzed the results of the clinical trials NCT04451096, NCT03377322, and NCT02459717 plus an additional study [188] and concluded that there is insufficient evidence supporting the use of probiotics to treat constipation in patients with PD; however, it also recognized that, according to the limited evidence available, probiotics have potential value in the treatment of PD-related constipation [189].

Fecal transplantation is based on the implantation of fecal content from a healthy donor into the GI tract of a recipient patient, thus changing the recipient’s intestinal microbiota and normalizing its composition, thereby providing therapeutic benefits [190]. Following the beneficial effects observed in the clinical setting for several GI and extraintestinal diseases, fecal transplantation has been considered as a feasible approach for the treatment of PD (for review, see Dutta et al. 2019 [58]). Supporting this view, preclinical evidence indicated that fecal transplantation elicited neuroprotective actions and attenuated the loss of dopaminergic neurons and the occurrence of motor alterations in a murine model of PD [191]. However, the clinical data are still scarce. A case report exploring the use of fecal transplantation in a PD patient with constipation showed improvement in bowel habits and PD-associated motor alterations for up to 2 months after the transplant, thus strongly supporting the potential of fecal transplantation [192]. A recent publication reported preliminary data on 15 patients with PD, and the results obtained in that study indicate that, in all cases, disease-related indices (both motor and non-motor) were improved, with an acceptable safety profile (mild and self-limiting side effects reported by five patients), after fecal transplantation [193]. Currently, seven clinical trials assessing the efficacy of fecal transplantation on PD are registered (ClinicalTrials.gov Identifiers: NCT05204641, NCT04837313, NCT03808389, NCT03876327, NCT03026231, NCT03671785, and NCT04854291); one of them has been withdrawn (NCT03026231), and only one is reported as completed (NCT03876327), although no data have been released.

## 5. Lipidomics as a New Tool to Identify Biomarkers in Parkinson’s Disease

Lipidomics is a research tool to study lipid pathways involved in several diseases, including those affecting the GI tract [194]. Lipidomics is a recent approach to find new biomarkers and to monitor patients with PD.

Lipids play roles in different biological mechanisms; they are the main constituents of cellular membranes and may anchor proteins. They even participate in cellular signaling and may act as transport molecules [195,196]. Importantly, lipids participate in most of the processes associated with PD pathogeny (oxidative stress, endosomal–lysosomal function, endoplasmic reticulum stress, immune response) and in PD etiology.

In this section, we will review the most relevant publications in the field and the state of the art of lipidomics in PD, beginning with some explanations about the different approaches in lipidomics and the principal lipid species that are implicated in PD.

### 5.1. Lipidomics Approaches

Lipidomics is a part of metabolomics that has recently become an independent omic. The use of this technique makes it possible to identify lipids from a biological sample [197]. These lipids can be potential biomarkers of disease. Interestingly, lipidomics can be used to obtain both qualitative and quantitative results, depending on the specific experimental design.

Due to the complexity of lipid species, it is necessary to combine different separation methods. In lipidomics, there are untargeted and targeted strategies depending on the outcome.

The untargeted strategies are: (1) shotgun lipidomics, which use a direct infusion, and there is a constant concentration of lipid solution; this methodology is usually combined with quadrupole-time of flight (Q-TOF) mass spectrometry (MS) [197]; and (2) liquid chromatography–mass spectrometry (LC-MS), in which the concentration of lipid solution is not constant, with high accuracy in the identification of individual lipid species; this technology may separate isomeric lipids, and it has a high separation efficiency and reproducibility [198].

In contrast, targeted lipidomics are focused on the quantification of predefined lipids. The preferable technique in this case is shotgun lipidomics as triple-quadrupole (QQQ) MS [199].

### 5.2. Lipids and Parkinson’s Disease

Lipids are classified as fatty acyls, glycerolipids, glycerophospholipids, sphingolipids, sterols, prenols, saccharolipids, and polyketides [199]. Here, we summarize the principal subclasses of lipids that seem to be involved in PD.

#### 5.2.1. Fatty Acyls

As briefly summarized in Table 4, there are different fatty acids, according to the presence and location of double bonds in the carbon chain. Humans can synthesize fatty acyls, but some of them need to be obtained from the diet.

An increase of saturated fatty acids (SFAs) could exacerbate PD, and higher levels of SFAs have been observed in the frontal cortex of PD patients [200]. Moreover, α-synuclein modulates the intake of palmitic acid (16:0) into the brain, and the accumulation of α-synuclein in PD brains could lead to increased central SFA levels [201].

Monounsaturated fatty acid (MUFAs) intake seems not to increase the risk of PD. Some MUFAs, such as oleic acid (18:1), could facilitate the interaction of α-synuclein with lipid rafts [202].

Polyunsaturated fatty acids (PUFAs) have been more studied than MUFAs in association with PD, but there is no agreement on the impact of PUFAs, although in PD animal models omega-3 PUFAs show a neuroprotective role [203].

SCFAs (less than 6C atoms such as acetate, 2:0; propionate, 3:0; and butyrate, 4:0), particularly those generated by microbial metabolism, and the relative proportion among different molecules, seem to be important in PD pathogenesis, including disease generation and progression (see above) [143,159,170,177]. As mentioned above (Section 4), these lipids are also important modulators of colonic motility [178]. However, despite the potential biological significance of these molecules, no lipidomic studies have addressed, as of today, their role in PD.

Another important lipid class are eicosanoids, a family of bioactive fatty acyls that play a role in inflammation [204]. Among them, prostaglandin (PG) E2 is dependent of its receptor, and it seems that increased levels of PG E2 play a role in animal and cellular PD models in a time-, location-, phenotype-, and receptor-dependent manner [205,206].

Carnitine can associate with fatty acids forming acyl-carnitines. Acyl-carnitines are involved in neurotransmission and apoptosis and are decreased in plasma of PD patients. Accordingly, acyl-carnitines have a protective role in PD models [207].

#### 5.2.2. Glycerolipids, Glycerophospholipids, and Sphingolipids

Glycerolipids also have been studied in PD, as the monoacylglycerol 2-arachidonoylglycerol (2-AG), which is an endocannabinoid ligand. Endocannabinoids have been linked to PD [208]. So far, studies about endocannabinoids and PD are inconclusive, and further investigation is necessary.

One of the most abundant glycerophospholipids is phosphatidylcholine (PC), which is involved in many structural roles. Decreased levels of PC have been observed in the plasma of PD patients [209].

Among sphingolipids, sphingosine-1 phosphate (S1P) is a bioactive lipid that promotes cell survival and induces the formation of α-synuclein, but it is protective in animal and cellular models of PD. Despite this evidence, further studies are necessary regarding these associations [210,211].

#### 5.2.3. Sterols

Sterols, such as cholesterol, have been widely studied in PD. Lower plasma cholesterol levels are associated with PD in males of more than 55 years old, whereas a high total cholesterol plasma level has been associated with increased risk of PD in patients between 25 and 54 years old [212].

### 5.3. Lipidomics Analysis in Parkinson’s Disease

Until now, treatments for PD have been unable to impede the disease progression. The identification of molecular changes early during the pathology may help to stop its progression. Data on lipid pathways have shown the importance of analyzing lipid species in PD more than lipid classes as a whole [213]. Therefore, the study of the lipidome using, whenever possible, minimally invasive samples could bring new biomarkers for prognosis and diagnosis of PD and even could serve for monitoring this pathology. Table 5 summarizes the main results described herein.

α-synuclein expressed in the brain interacts with phospholipids (PLs) and fatty acids. Recent studies have shown that α-synuclein preferentially binds specific lipids and these bindings enhance the aggregation and interfere with catalytic lipid enzymes, affecting lipid metabolism. Therefore, interaction of α-synuclein with lipids is important in homeostasis, and PD-associated mutations could underlie the pathological state [213]. In this line, Fanning et al. (2019) performed an unbiased lipidomic analysis to study the relation between α-synuclein and lipid metabolic pathways. Oleic acid and other MUFAs’ metabolism induces neurotoxicity increasing α-synuclein, thus highlighting the importance of studying this lipid class in relation with PD [219].

Most of the related literature about PD is focused on providing new targets for early interventions in PD. Farmer et al. (2015) used animal models of PD to study lipid alterations [216]. To induce PD, the authors used a low dose of 6-OHDA in rats. The authors performed high-performance liquid chromatography electrospray ionization tandem mass spectrometry (HPLC-ESI-MS/MS) to profile PCs and sphingolipids of substantia nigra. In total, 115 lipids were identified, and 19 lipid species were downregulated. PC lipid species were downregulated in animals treated with 6-OHDA, reflecting some degree of structural rearrangement. On the other hand, LPCs, important for neuroinflammatory signaling, were upregulated [216]. LPCs are lipids synthesized from PCs via phospholipase A2 (PLA2). The increase of PLA2 activity has been associated with apoptotic effects [230], and it has been reported that 6-OHDA induces PLA2 activity within the nigrostriatal tract [216,231]. More recently, a lipidome analysis using ultrahigh-performance liquid chromatography combined with high-resolution mass spectrometry (UHPLC-MS/MS) was used to evaluate changes in the lipidome of the midbrain after paraquat exposure in mice [227]. In this study, the authors found 35 significantly elevated lipids and 18 remarkably decreased lipids. In particular, proinflammatory lipids, such as ceramide, lisophosphatidylcholine (LPC), lysophosphatidylserine (LPS), and lysophosphatidylinositol (LPI), were increased, whereas sphingomyelin (SM) was decreased, suggesting ceramide metabolism is disturbed in paraquat-exposed mice. Interestingly, these findings correlated well with motor deficits and alterations in mRNA expression of genes involved in ceramide metabolism in the midbrain. Furthermore, the authors found elevated levels of proinflammatory cytokines in the serum of both mice and humans exposed to this pollutant, suggesting an occurrence of systemic inflammation underlying paraquat-induced neurotoxicity [227].

6-OHDA has also been used in cellular models such as the neuroblastoma cell line SH-SY5Y. Recently, Xicoy et al. (2020) performed an in vitro study to explore the effect of 6-OHDA on the lipidome of the cell line SH-SY5Y [222]. LC-MS analyses were performed for lipidomics analysis, and 306 PLs were identified. There were changes in PC, phosphatidylglycerol, phosphatidylinositol, phosphatidylserine, sphingomyelin, and total cholesterol in the cells treated with 6-OHDA. The results supported the validation of cultured 6-OHDA-treated SH-SY5Y cells as a cell model for in vitro studies of PD [222].

Human tissue of PD patients can also be used for lipidomics analysis. Cheng et al. (2011) collected samples from 10 PD patients and 10 healthy controls and analyzed primary visual cortex, amygdala, and anterior cingulate cortex tissues using LC-MS for an initial screening of 200 lipids [214]. There were significant differences in 79 lipids between sphingolipids, glycerophospholipids, and cholesterol, and 73 lipids were significantly changed in the visual cortex. GC-MS analysis showed that six oxysterols were increased in the PD visual cortex. The most relevant conclusion was that there were changes in the lipid metabolism of the visual cortex in PD before the manifestation of clear symptoms of PD; therefore, lipid metabolism analysis in the visual cortex could represent a novel strategy to facilitate early treatment [214]. In a recent study, UHPLC-MS was used to study post-mortem frontal and cingulate cortex, as well as cerebrospinal fluid (CSF). Extracellular vesicles (EVs) were evaluated due to their important role in systemic inflammation. The authors found that sphingolipid levels are altered in both brains and EVs from patients with LB disorders (which encompass PD, PD dementia, and dementia with LB), irrespective of the co-occurrence of glucocerebrosidase (GBA) gene mutations. EVs carried ceramides and neurodegeneration-linked proteins (alpha-synuclein and tau) whose interaction induced α-synuclein aggregation, suggesting that EVs are involved in disease propagation [226].

Biomarkers derived from biological samples obtained with noninvasive or minimally invasive procedures, such as plasma, represent a significant advantage. In this sense, some studies in PD have worked following this approach in the lipidomics field, but publications using plasma for lipidomics studies in PD are still relatively scarce.

There are some PD animal models in which investigators studied the lipidomic profile in plasma. Tyurina et al. (2015) performed LC/MS analysis of cardiolipins in the substantia nigra and plasma of rotenone-treated rats [215]. Rotenone acts as an inhibitor of mitochondrial respiratory complex I and develops in vivo neuropathological features of PD. To assess oxidative metabolism of the mitochondria-specific phospholipid cardiolipin, the authors performed oxidative lipidomics. Rats exposed to rotenone showed elevated levels of PUFA cardiolipins in plasma [215]. Since polyunsaturated PLs are the major substrates for oxidation and, in normal conditions, cardiolipin is in the inner mitochondrial membrane [215,232], this observation suggests that mitochondrial dysfunction and oxidative stress are important contributors to neural loss and the development of PD [233]. Accumulation of this phospholipid produces brain injury, and degeneration of dopaminergic neurons has been associated with mitochondrial dysfunction [234]. The exposure to rotenone causes depletion of PUFA cardiolipin and elevated levels of cardiolipins in plasma. Detection of these cardiolipins in plasma may lead to the development of new biomarkers of mitochondrial dysfunction associated with PD [215].

Some studies in plasma using LC/MS reported an implication of lower glucocerebrosidase activity with PD risk. Here, we collected two of them [209,217]. First, Chan et al. (2017) analyzed the lipidomic profile of plasma from 150 PD patients and 100 controls. They carried out the lipidomic analysis using LC/MS and reported 520 plasma lipid species from 39 lipid subclasses. Triacylglycerides (TGs) and monosialodihexosylganglioside (GM3) were different between PD and control participants. The results obtained by these authors suggest that elevated GM3 levels in plasma were associated with PD [217]. Second, Zhang et al. (2017) analyzed the lipidomic profile in the plasma of 170 PD patients and 120 controls using HPLC-MS. The results yielded 503 plasma lipid species of 34 subclasses of lipids. The authors observed differences in the plasma concentration of TGs and ganglioside-NANA-3 between PD patients and controls. Elevated concentration of ganglioside-NANA-3 is associated with PD. This lipid species is related to the metabolic pathway of glucosylceramide, which is linked to PD [209]. Another study used LC-MS/MS to evaluate plasma lipidomics in individuals with different neurodegenerative diseases, including idiopathic PD, dementia with LB and multiple system atrophy, as synucleinopathies, and Alzheimer’s disease and progressive supranuclear palsy, as tauopathies [228]. In this study, the authors found that the plasma levels of sphingosine-1-phosphate (which seems to have a neuroprotective role against aggregate formation) were lower, and those of monohexylceramide and lactosylceramide (which seem to be involved in neuroinflammation and neuronal cell death) were higher in the neurodegenerative disease groups, compared with the healthy controls, supporting the idea that abnormal sphingolipid metabolism has a key role in neurodegeneration [228].

Nuclear magnetic resonance (NMR) spectrometry is another approach to determine a lipid profile. This approach is rarely used in omic PD studies because of the restricted number of specimens that can be measured using this technique. Pizarro et al. (2019) applied the NMR lipidomic approach in plasma to separate between 38 early-stage and 10 advanced-stage PD patients, 23 patients with Alzheimer’s disease, and 23 healthy controls [221]. These authors only studied lipoproteins in human plasma with this method. The dataset was divided into 86 training and 8 external test samples for validation. Thirty chemical shift buckets enabled differentiation between PD patients, Alzheimer’s disease patients, and controls, demonstrating that this approach is a good tool for PD diagnosis [221].

Some studies have selected skin fibroblasts as a model of primary human cells to investigate the fibroblasts lipidome as a biomarker source for early diagnosis in PD. Calvano et al. (2019) performed lipidomics analysis based on hydrophilic interacting liquid chromatography coupled to electrospray ionization and mass spectrometry (HILIC/ESI-MS) [220]. Then, PLs from fibroblasts of five PD patients and healthy controls were characterized by single and tandem MS identifying more than 360 PLs. The PD group showed an abnormality of PL metabolism, highlighting that PLs as phosphatidylcholines (PCs) and phosphatidylethanolamines (PEs) could be feasible biomarkers of PD. Plasmalogens are important markers of oxidative stress, and they are required for the correct function of membrane proteins. Ethanolamine plasmalogens were dysregulated in PD, pointing to an association with neurodegeneration [220].

Lobasso et al. (2017) used skin primary fibroblasts to study parkin mutations that are considered to be a cause of early-onset PD. They studied the lipid alterations associated with the lack of parkin protein. They analyzed the lipidome of fibroblasts performing a matrix-assisted laser desorption/ionization-time-of-flight mass spectrometry (MALDI-TOF/MS) [218]. PLs and glycosphingolipids were altered in parkin mutant fibroblasts and lysophosphatidylcholines (LPC) were increased, pointing to this molecule as a marker of the neuroinflammatory state [218].

Recently, Sinclair et al. (2021) used metabolomics to search for sebum lipids in PD [225]. Sebum is a biofluid that can be obtained noninvasively. The authors used LC/MS to analyze metabolites from 80 drug-naïve PD patients, 138 medicated PD patients, and 56 healthy controls [225]. Partial least squares–discriminant analysis (PLS-DA) models were performed and validated using bootstrap resampling with replacement. Variable importance project (VIP) scores were calculated to define features responsible for the variance in PLS-DA, and then receiver operating characteristic (ROC) curve analysis was performed for those variables with a VIP score higher than 1. Sensitivity and specificity were below 60% for medicated PD and naïve PD. VIP scores confirmed 10 common variables between these two groups and were evaluated further. Multivariate ROC curves for each common variable yielded higher area under the curve (AUC) values. Analysis showed that carnitine shuttle is the most important pathway linked to drug-naïve PD patients [225]. Decreased acyl-carnitine associated with insufficient β-oxidation is a potential diagnostic marker for PD [207]. Following these results, sphingolipids may work as biomarkers of PD; perturbations in their pathway have been linked to defects in lysosomal and mitochondrial metabolism, which are involved in PD [235]. The results revealed a different composition of sebum between PD patients and controls. The overlap of 10 metabolites from separate statistical analyses for drug-naïve PD and medicated PD pointed to the evidence that these compounds are associated with PD and not with dopaminergic treatment [225]. Overall, these data indicate that skin sebum may be a good biological sample for finding new lipid biomarkers of PD.

Lately, combined omics has been used to investigate PD. In this regard, Gill et al. (2020) used high-resolution LC-MS, metabolomics, and lipidomics to investigate the metabolic changes with a novel T-cell vaccination in PD mice [223]. In this case, stool samples were collected from three PD-induced mice and three vaccinated PD mice for global metabolomics and lipidomics. L-carnitine and butyrobetaine were involved in lipid metabolism, and both were elevated in vaccinated mice. L-carnitine seems to act as a neuroprotector. Diacylglycerols and TGs were upregulated in the PD-induced mice. Metabolites involved in immune response were elevated in vaccinated mice, and TGs were downregulated [223]. These observations support the view that targeting the adaptative immune system in PD is a very interesting strategy to be explored in detail in future studies.

Recently, lipidomics and transcriptomics have also been studied together in samples of substantia nigra and putamen in PD. Xicoy et al. (2020) performed a combined lipid profile, using LC/MS, and RNA sequencing in post-mortem samples, from 10 PD patients and 10 controls. The study focused only on PLs and sphingolipids. In total, 269 PLs were identified and 39 upregulated and 41 downregulated transcripts [224]. Analysis revealed that a group of genes associated with the lipid classes were differently expressed in the substantia nigra and putamen. The results pointed to a connection between lipidome and transcriptome; in fact, transcriptome changes that lead to differences in the lipid profile could be a mechanism of neurodegeneration in PD. Some familial genes are known to modulate several lipid metabolism pathways. Interestingly, the authors concluded that some of the changes in the PD brain lipid profile were gender-dependent [224]. A similar conclusion was obtained in a machine-learning study that tried to identify PD severity markers using whole blood samples. The authors found 517 lipid species from 37 classes, including all major classes of glycerophospholipids, sphingolipids, glycerolipids, and sterols. The random forest machine-learning algorithm applied allowed the authors to demonstrate specific lipid classes (particularly, dihydro sphingomyelin, plasmalogen phosphatidylethanolamine, glucosylceramide, and dihydro globotriaosylceramide) interrelated in a sex-dependent manner with the degree of the severity of motor symptoms [236].

Lipidomics have also been combined with the analysis of the metallome (comprehensive analysis of the entirety of metal and metalloid species within a cell or tissue type). In this study, *C. elegans* was exposed to redox-active metals, and modern hyphenated techniques, such as capillary electrophoresis coupled to inductively coupled plasma mass spectrometry and ultrahigh-performance liquid chromatography mass spectrometry, were used. Acute exposure to iron and manganese and chronic exposure to zinc altered phospholipid composition, with an impact on the plasma membrane, changing also its fluidity and permeability; acute iron also significantly altered cardiolipins in the inner mitochondrial membrane. Thus, these authors showed lipidomic alterations as a consequence of altered metallostasis due to metal treatment [229].

As described here, most studies regarding lipidomics in PD use LC/MS approaches in animal or cell models. Some studies collected human tissues from PD patients and controls, and a few of them have done the analysis in plasma, but none in stool (the only study using stool was performed in mice [223]). Multi-omics seems to be the future tool to search for biomarkers of PD. There are also new approaches that require further investigation such as targeting the adaptative immune system.

Thus, lipidomics is a promising approach for finding biomarkers of PD because of the implication of lipid species in the development of the disease and the changes in the lipidome of PD patients, not only compared to healthy controls but also among the different stages in the progression of the disease.

### 5.4. Microbiota, Probiotics, and Lipidomics in Parkinson’s Disease

As shown above, lipidomic analysis in PD represents a new approach to finding biomarkers of this disease and monitoring it. In particular, it is necessary to go deeper in the study of biomarkers in the early stage of PD and establish the risk of developing PD because there is a gap in the knowledge about the risk and early diagnosis of PD.

Since the gut microbiota seems to be involved in the development of PD, and this microbial community produces specific metabolites, including lipid species, lipidomic research focused on the gut microbiota may constitute a new key approach to the PD problem and its GI complications. Thus, identification of lipid biomarkers derived from gut microbiota, obtained noninvasively from the feces, could be extremely useful to help in the early diagnosis of PD and to determine to what extent gut dysbiosis precedes or occurs after the development of GI prodromal symptoms. However, as stated above, only one lipidomic study has been performed using stool samples, and these were obtained from mice [223]. On the other hand, the relationship of gut microbiota and constipation in PD has also been addressed, and some studies indicate that the reduced production of SCFA may indeed be involved in this problem, but the approach used was through microbiota metagenomics, not through lipidomics itself [178]. Obviously, many more studies are needed to clarify this.

Taking into account the relevant role of the intestinal microbiota in the pathogenesis of PD and the growing interest in its manipulation, mainly with probiotics, as a therapeutic option (see above), one of the most attractive fields to study is the metabolites that probiotics produce in the host and their potential biological functions as disease modifiers. Recently, metabolomics and lipidomics have been considered as tools to study probiotics and the metabolic changes that they may provoke in the host. Following in vitro observations [237,238,239], several lipidomic studies have consistently shown that probiotics can affect in a selective manner, with both probiotic- and host-related selectivity, lipid-related metabolic profiles in the host, with changes affecting, for instance, SCFAs, bile acids, and other lipid-related metabolites (for review, see [240]). Accordingly, probiotics have been shown to affect, in a selective manner, the lipidomic profile in several animal models, including *C. elegans* and rodents [239,241,242,243], as well as in humans [244,245]. Despite these data and the indications that probiotics might be beneficial in PD (see above), no lipidomic studies associated with probiotic treatment have been performed in relation to PD. There is still a significant gap in this field that needs to be filled in the near future.

There is a strong link between gut microbiota and lipids from bacteria metabolism, and lipidomics may provide new data to pave the way in the study of gut microbiota and their lipid metabolism in PD. In particular, lipidomics used for the study of probiotics in the PD context will provide significant advances in the investigation of PD, helping to understand its pathophysiology, the identification of potential biomarkers and therapeutic targets, and the development of new treatments.

## 6. Conclusions

PD is a complex disease with severe and debilitating symptoms affecting motor and sensory performance as well as the GI tract, which mainly displays motor alterations in these patients. Although neuronal degeneration, associated with α-synuclein aggregation within the CNS, seems to be the main pathological feature, this process is likely initiated within the GI tract. In both humans and several animal models (with genetic alterations or induced by neurotoxin administration), it has been demonstrated that α-synuclein aggregation occurs within the ENS and is associated with GI motility alterations, well before PD motor symptoms are evident.

Importantly, PD motor and GI alterations have been suggested to be associated to changes in the intestinal microbiota. However, while numerous studies have demonstrated the presence of dysbiosis in PD patients, there is no consensus as it relates to the alterations presented or the causal effect, with dysbiosis being regarded as either a cause or a perpetuating consequence of the disease. In any case, given the consistent dysbiotic state, the microbiota has emerged as a target for the treatment of the disease, with two main approaches: use of probiotics and fecal transplant. The supporting evidence, particularly the one related to fecal transplant, is still very limited, and additional controlled clinical trials are necessary. Moreover, probiotics have been investigated to treat GI motor disturbances (constipation) associated with PD. Currently, there is insufficient evidence supporting the use of probiotics to treat constipation in patients with PD; however, according to the limited evidence available, probiotics have potential value in the treatment of PD-related constipation.

Several studies indicate that changes in lipid metabolism, including host and microbial metabolism, also might be important in PD pathogenesis. In this regard, lipidomics has emerged as a relevant field in PD, particularly as it relates to the discovery of lipid metabolic-related biomarkers, which can be used to monitor disease progression and severity as well as treatment efficacy. However, its application to the study of lipid metabolites derived from the gut microbiota, particularly as a way to find new early biomarkers of PD, has yet to be done. Furthermore, the interaction between probiotic-dependent microbiota and lipid metabolism, as assessed through lipidomics, is emerging as an interesting field of study for multiple diseases. However, this relationship has not been addressed in the case of PD, and there remains an important gap of knowledge to be filled in the near future, which may provide new approaches to solve the PD puzzle.

## Figures and Tables

**Table 1:** Summary of the main characteristics of the neurotoxin-induced PD models.

Neurotoxin	Pathogenesis	PD Symptoms	Advantages	Disadvantages
6-OHDA	Loss of DA innervation	Rotational motor behavior Sensory motor deficits Akinesia (with bilateral administration)	Activation of glial cells Economical Different variants depending on site and dose administered	Needs intracerebral administration Does not mimic the multisystem pathology of PD Does not induce a progressive nigrostriatal degeneration Does not induce LB formation nor synuclein aggregation
MPTP	Dose-dependent loss of DA neurons Reduced DA levels in striatum and midbrain DA neuron loss	Dose-dependent locomotor alterations Sensorimotor deficits	Translatable to human disease Easy to administer	Not toxic in rats Does not induce LBs Does not mimic the multisystem pathology of PD Variability in behavioral and biochemical results High mortality
Rotenone	DA neuron loss in SNpc Nigrostriatal dopaminergic denervation	Motor disturbances Sensory-motor deficits	Easy to administer Induces most motor symptoms of PD	Toxicity in other organs Causes nonspecific cerebral damage High mortality High interindividual variability
Paraquat	Controversy across studies Recent studies describe a reduction in dopaminergic neurons in the SNpc	Decreased locomotor activity and sensory-motor function	Easy to administer Translatable to human disease Increased synuclein immunoreactivity and LB-like structures in DA neurons of the SNpc Increased synuclein immunoreactivity in enteric neurons	Controversial results Important toxic effects, which can difficult the interpretation of behavioral tests High mortality (if using high doses)

Abbreviations: 6-OHDA, 6-hydroxydopamine; DA, dopamine; LB, Lewy bodies; MPTP, 1-methyl-4-phenyl-1,2,3,6-tetrahydropyridine; PD, Parkinson’s disease; SNpc, substantia nigra pars compacta.

**Table 2:** Changes in the intestinal microbiota of PD patients at the phylum and family levels ^1^.

Level	Group	Change	References
Phylum	Actinobacteria Bacteroidetes Firmicutes Lentisphaera Synergistetes Verrucomicrobia Proteobacteria	↑	[118,143,144]
	Firmicutes	↓	[143]
Family	*Acidaminococcaceae* *Akkermansiaceae* *Barnesiellaceae* *Bifidobacteriaceae* *Bradyrizobiaceae* *Christensenellaceae* *Clostridiales Family XIII. Incertae Sedis* *Clostridialesvadin BB60 group* *Corpobacillaceae* *Corynebacteriaceae* *Coriobacteriaceae* *Desulfovibrionaceae* *Enterobacteriacea* *Enterococcaceae* *Erysipelotrichaceae* *Eubacteraceae* *Lachnopsiraceae NK4A* *Lactobacillaceae* *Porphyromodaceae* *Odoribacteriaceae* *Oscillospiraceae* *Porphyromonadaceae* *Prevotelaceae* *Rikenellaceae* *Ruminococacceae* *Streptococcaceae* *Synergistaceae* *Tissierellaceae* *Thermoanaerobacterales Family IV. Incertae Sedis* *Unclassified Victivallales* *Veillonellaceae* *Verrucomicrobiaceae*	↑	[118,132,134,136,137,139,141,143,144,145,146,147,148,149,150,151,152]
	*Corpobacillaceae* *Enterococcaceae* *Erysipelotrichaceae* *Hungateiclostridiaceae* *Lactobacillace* *Muribaculaceae* *Pasteurellaceae* *Peptostreptococcaceae* *Porphyromonadaceae* *Puniceicoccaceae* *Prevotellaceae* *Rikenellaceae* *Ruminococcaceae* *Streptococcaceae* *Uricibacteraceae* *Verrucomicrobiaceae*	No change	[118,129,132,136,139,140,141,143,146,149,150,151,152,153,154,155]

^1^ The nomenclature used for the bacterial groups corresponds to that used in the original reports referenceed. Arrows indicate an increase (↑) or a decrease (↓) in the intestinal microbiota at the phylum and family level.

**Table 3:** Main changes described in the intestinal microbiota of PD patients (taxa level below family) ^1^.

Bacterial Group	Change	References
*Acidaminococcus*	↑	[118,139]
*Akkermansia*	↑	[118,146,148,151,153,154]
*Alistipes*	↑	[118,136,144]
*Alistipes shahii*	↑	[154]
*Anaerofustis*	↑	[139]
*Anaerotruncus*	↑	[139,156]
*Aquabacterium*	↑	[156]
*Bacteroides*	↓	[137,139,147]
*Bacteroides fragilis*	↓	[157]
*Bacteroidetes* spp.	↑	[153]
	↓	[132]
*Bifidobactium* spp.	↑	[118,132,143,144,146,150]
*Bilophila* spp.	↑	[129,144]
*Blautia* spp.	↓	[139,150,153,158]
*Butyricicoccus* spp.	↑	[156]
	↓	[129,140]
*Butyricimonas*	↑	[118,136]
*Butyrivibrio*	↓	[118]
*Campylobacter*	↑	[139]
*Catabacter* spp.	↑	[147]
*Citrobacter*	↑	[139]
*Cloacibacillus*	↑	[118]
*Coprococcus* spp.	↓	[153]
*Clostridiales incertae sedis IV*	↓	[145]
*Clostridium IV*	↑	[156]
*Clostridium XVIII*	↑	[156]
*Clostridium XIVa*	↓	[150]
*Clostridium coccides*	↓	[157]
*Clostridium leptum*	↓	[157]
*Clostridium saccharolyticum*	↓	[154]
*Collinsella*	↑	[118,144]
*Dehalobacterium*	↑	[139]
*Desulfovibrio*	↑	[118,144]
*Dorea* spp.	↓	[139,147,153]
*Enterococcus*	↑	[158]
*Escherichia*	↑	[118,158]
*Eubacterium*	↓	[154]
*Eubacterium biforme*	↓	[154]
*Faecalibacterium*	↓	[118,139,143,147,158]
*Faecalibacterium prusnitzii*	↓	[132]
*Finegoldia*	↑	[139]
*Fusicatenibacter*	↓	[140]
Fusobacteriales (unclassified)	↑	[118]
*Hallomonas*	↑	[139]
*Holdemania*	↑	[156]
Hungatella	↑	[118]
*Hydrogenonaerobacterium*	↑	[137]
*Kliebsiella*	↑	[141]
*Lactobacillus (nomenclaure updated to Lacticaseibacillus)*	↓	[118,155,156]
	↑	[143,144,146,147,150,152,157]
*Lactococcus*	↓	[155]
	↑	[141]
*Mahella*	↑	[118]
*Megasphaera*	↑	[118]
*Methanobrevibacter*	↑	[139]
*Methanomassiliicoccus*	↑	[139]
*Mogibacterium*	↑	[118]
*Mucispirillum*	↑	[152]
*Oscillibacter*	↑	[144]
*Oscillospira*	↑	[147,151,153]
*Parabacteroides*	↑	[136,152]
*Peptoniphilus*	↑	[139]
*Phascolarctobacterium*	↓	[139]
*Porphyromionas*	↑	[152]
*Prevotella*	↓	[118,132,136,137,139,145,147,150,152,154,155,157]
	↑	[143,148]
*Prevotella copri*	↓	[154]
*Proteus* spp.	↑	[129,158]
*Pseudoramibacter_Eubacterium*	↑	[139]
*Roseburia* spp.	↓	[118,139,141,144,150,151,153]
	↑	[129]
*Ruminococcus*	↓	[139,151,158]
*Sediminibacterium*	↓	[156]
*Sphingomonas*	↑	[156]
*Streptococcus*	↑	[118,158]
*Sunergistes*	↑	[139]
*Sutterella*	↓	[139]
*Turicibacter*	↑	[139]
*Varibaculum*	↑	[139]
*Veillonella*	↑	[118]
Victivallales (unclassified)	↑	[118]

^1^ The nomenclature used for the bacterial groups corresponds to that used in the original reports referenced. The latest updates in nomenclature are indicated, as appropriate, in the table. Arrows indicate an increase (↑) or a decrease (↓) in the intestinal microbiota.

**Table 4:** Fatty acids.

Type	Definition	Example	Abbreviation	Number of C Atoms
Saturated	No C-to-C double bounds	Palmitic acid	16:0	16 C atoms and 0 C-to-C double bound
Unsaturated	C-to-C double bounds			
Monounsaturated (MUFA)	One C-to-C double bound	Oleic acid	18:1	18 C atoms and one C-to-C double bound
Polyunsaturated (PUFA)	Two or more C-to-C double bounds	Linoleic acid (omega-6)	18:2	18 C atoms and two C-to-C double bounds
		Linolenic acid (omega-3)	18:3	18 C atoms and three C-to-C double bounds

**Table 5:** Lipidomics studies in Parkinson’s disease.

Author and Year of Publication	Lipidomic Approach	Biological Samples	Conclusions
Cheng et al. 2011 [214]	LC-MS	10 PD and 10 HC. Primary visual cortex, amygdale, and anterior cingulated cortex tissues.	Changes in lipid metabolism occur in PD visual cortex in the absence of obvious pathology. Lipid metabolism in the visual cortex may represent a novel target for treatment of non-motor symptoms.
Tyurina et al. 2015 [215]	LC-MS	Rats exposed to rotenone	Elevated levels of PUFA cardiolipins in plasma.
Farmer et al. 2015 [216]	HPLC-ESI-MS/MS	Rats treated with 6-OHDA	Upregulation of lysophosphatidylcholine, important for neuroinflammatory signaling.
Zhang et al. 2017 [209]	HPLC-MS	Human plasma (170 PD; 120 HC)	Elevated concentration of ganglioside-NANA-3 is associated with PD.
Chan et al. 2017 [217]	LC-MS	Plasma: 150 idiopathic PD and 100 HC	Elevated GM3 levels in plasma might be associated with PD.
Lobasso et al. 2017 [218]	MALDI-TOF-MS	Primary skin fibroblasts	Phospholipids and glycosphingolipids were altered in parkin mutants’ fibroblasts and lysophosphatidylcholine were increased, pointing to this molecule as a marker of neuroinflammatory state.
Fanning et al. 2019 [219]	LC-MS	Rat cortical neurons Human iPS-derived neurons Yeast cells	Monounsaturated fatty acid metabolism induces neurotoxicity increasing α-synuclein.
Calvano et al. 2019 [220]	HILIC/ESI-MS MS	Primary skin fibroblasts (5 PD and HC)	Abnormal PLs metabolism and plasmalogens in PD could be associated with neurodegeneration.
Pizarro et al. 2019 [221]	NMR	Plasma (38 early-stage PD, 10 PD-related dementia, 23 Alzheimer’s dementia, and 23 HC9)	30 chemical shift buckets enabled differentiation between PD patients, Alzheimer patients, and controls, demonstrating that this approach is a good diagnostic tool for PD.
Xicoy et al. 2020 [222]	LC-MS	Neuronal cell line SH-SY5Y treated with the neurotoxin 6-hydroxydopamine.	Changes in phosphatidylcholine, phosphatidylglycerol, phosphatidylinositol, phosphatididylserine, sphingomyelin, and total cholesterol in 6-OHDA-treated cells.
Gill et al. 2020 [223]	UHP-LC-MS. Metabolomics and lipidomics.	Stool samples (3 PD mice and 3 vaccinated (T-cell vaccination) PD mice)	L-carnitine seems to act as neuroprotector. Diacylglycerols and triacylglycerols were upregulated in the PD mice. Targeting the adaptative immune system in PD might have potential therapeutic value.
Xicoy et al. 2020 [224]	LC-MS and RNAseq. Lipidomics and transcriptomics.	Susbtantia nigra and putamen from post-mortem samples (10 PD and 10 HC)	Transcriptome changes leading to differences in the lipid profile could be a mechanism of neurodegeneration in PD. Some of the changes in the PD brain lipid profile were gender dependent.
Sinclair et al. 2021 [225]	LC-MS	Skin sebum (80 drug naïve PD patients, 138 medicated PD patients and 56 HC)	Alterations in carnitine shuttle, SP, arachidonic acid, and FA metabolism in PD. Skin sebum may work as a good biological sample to identify biomarkers of PD.
Kurzawa-Akanbi et al. 2021 [226]	UHP-LC-MS	59 samples of post-mortem frontal cortex and 48 samples of cingulate cortex from matched LBD and controls (with and without GBA mutations); 15 post-mortem CSF samples	Increase in ceramides in LBD post-mortem tissue and CSF, irrespective of GBA mutation status (although GBA mutations might increase risk). Marked parallel elevation of ceramide levels in EV, probably associated with ER stress, a general loss of endolysosomal homeostasis, and lysosomal degradation capacity. This might cause abnormal pathogenic α-synuclein to be associated with EV.
Tong et al. 2022 [227]	UHP-LC-MS	Midbrain of mice exposed to paraquat	Increase in proinflammatory lipids in midbrain, including ceramide, LPC, LPS, and LPI, and a decrease in SM.
Oizumi et al. 2022 [228]	LC-MS	Plasma samples (49 healthy controls, 58 idiopathic PD patients, 28 DLB patients, 13 MSA patients, 13 AD patients, and 16 PSP patients)	Compared with healthy controls, the plasma levels of S1P decreased, whereas those of monohexylceramide and lactosylceramide increased in patients with the neurodegenerative disease studied. Abnormal SP metabolism is key in neurodegeneration.
Blume et al. 2022 [229]	UHP-LC-MS. Lipidomics and metallomics	*C. elegans* exposed to iron, manganese, zinc …	Altered metallostasis due to metal treatment caused lipidomic alterations affecting phospholipid composition in plasma membrane and cardiolipins in the inner mitochondrial membrane.

Studies are presented in chronological order. Abbreviations: 6-OHDA, 6-hydroxidopamine; AD, Alzheimer’s disease; DLB, dementia with Lewy bodies; GM3, monosialodihexosyl ganglioside; FA, fatty acid; HC, healthy controls; HILIC/ESI-MS/MS, liquid chromatography coupled to electrospray ionization and mass spectrometry; HPLC-ESI-MS/MS, high-performance liquid chromatography electrospray ionization tandem mass spectrometry; HPLC-MS, high-performance liquid chromatography–mass spectrometry; LC-MS, liquid chromatography–mass spectrometry; LPC, lysophosphatidylcholine; LPI, lysophosphatidylinositol; LPS, lysophosphatidylserine; MALDI-TOF-MS, matrix-assisted laser desorption/ionization-time-of-flight mass spectrometry; MSA, multiple system atrophy; NMR, nuclear magnetic resonance; PD, Parkinson´s disease; PLs, phospholipids; PSP, progressive supranuclear palsy; PUFA, polyunsaturated fatty acids; SM, sphingomyelin; SP, sphingolipids; UHP-LC-MS, ultrahigh-performance liquid chromatography–mass spectrometry.

## Data Availability

No new data were created or analyzed in this study.
